# Exploration of an epoxidation–ring-opening strategy for the synthesis of lyconadin A and discovery of an unexpected Payne rearrangement

**DOI:** 10.3762/bjoc.9.132

**Published:** 2013-06-18

**Authors:** Brad M Loertscher, Yu Zhang, Steven L Castle

**Affiliations:** 1 Department of Chemistry and Biochemistry, Brigham Young University, C100 BNSN, Provo, UT, 84602, USA

**Keywords:** Lewis acid, lyconadin A, Myers alkylation, Payne rearrangement, Shi epoxidation

## Abstract

In the context of synthetic efforts targeting the alkaloid lyconadin A, scalemic epoxide **25** was prepared by a highly stereoselective sequence involving a Myers alkylation and a Shi epoxidation. Ring-opening of this epoxide with a vinylcopper complex afforded alcohol **26** instead of the expected product **27**. An unusual Lewis acid promoted Payne rearrangement of an α-trityloxy epoxide is proposed to account for this outcome.

## Introduction

**Figure 1:**
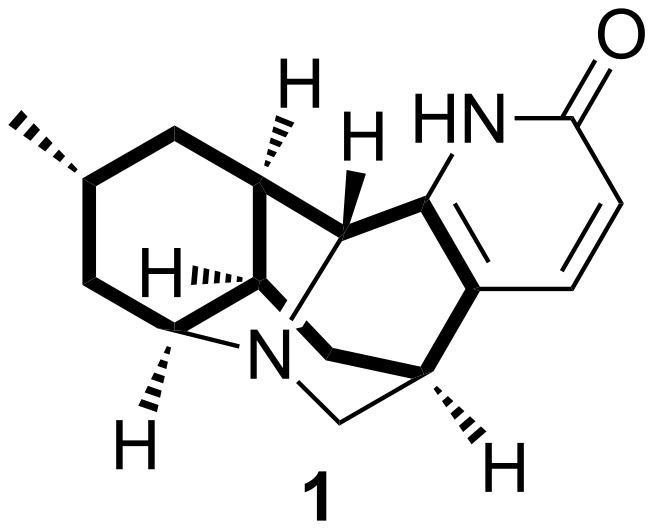
Lyconadin A.

Lyconadin A (**1**, Figure 1) was isolated from the club moss *Lycopodium complanatum* in 2001 by Kobayashi and co-workers [1]. Subsequent to this discovery, lyconadins B–F were isolated and characterized [2–4]. Biological assays revealed that **1** exhibits cytotoxicity against murine lymphoma L1210 and human epidermoid carcinoma KB cells (IC_50_ = 0.46 μg/mL and 1.7 μg/mL, respectively) [1]. Moreover, **1** has been shown to promote nerve growth factor biosynthesis in 1321N1 human astrocytoma cells [2]. In addition to its interesting bioactivity, lyconadin A presents a significant synthetic challenge by virtue of its unique pentacyclic skeleton, which contains six stereocenters and a pyridone ring. It is therefore not surprising that **1** has attracted the attention of the organic synthesis community. The first total synthesis of lyconadin A was reported in 2007 by Smith and Beshore [5–6], and efforts from the Sarpong [7–8] and Fukuyama [9–10] groups have also culminated in the construction of **1**.

Our initial interest in lyconadin A was sparked by recognition that a 7-*exo*–6-*exo* cyclization cascade would efficiently furnish its bicyclo[5.4.0]undecane system, which is shown in bold in Figure 1. Subsequent to this observation, we performed model studies that demonstrated the viability of highly stereoselective 7-*exo*-*trig* acyl radical–6-*exo*-*trig* alkyl radical cyclizations as a means of preparing bicyclo[5.4.0]undecanes fused to aromatic rings [11]. Then, we devised an annulation protocol inspired by the work of Donohoe and co-workers [12–13] that provided access to substituted pyridones of the type found in **1** from thioester precursors [14]. Based on these encouraging results, we decided to target lyconadin A for synthesis. Herein, we provide an account of our studies directed toward the construction of this alkaloid. Specifically, we describe our efforts to prepare advanced intermediates that could be employed in the aforementioned pyridone annulation and tandem radical cyclization processes. In the course of this work, we discovered an unusual Payne-like rearrangement process that occurred in preference to the ring-opening of a hindered epoxide.

## Results and Discussion

**Scheme 1:**
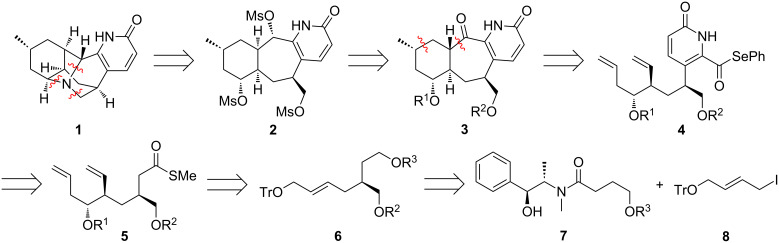
Retrosynthetic analysis of **1**.

Our retrosynthetic analysis of lyconadin A is shown in Scheme 1. We reasoned that **1** could be formed by an alkylation cascade triggered by exposure of trimesylate **2** or a related electrophile to ammonia. A sequential alkylation process would serve as a viable alternative in the event of problems with this approach. In turn, *cis*-fused trimesylate **2** could be derived from *trans*-fused tricyclic ketone **3** by epimerization and standard functional-group manipulations. Based on the aforementioned model study [11], 7-*exo*–6-*exo* tandem radical cyclization of phenyl selenoester **4** was expected to produce ketone **3**. Disassembly of the pyridone moiety of **4** according to our annulation protocol [14] revealed thioester **5** as a suitable precursor. We believed that this compound could be prepared from alkene **6** in two consecutive epoxidation–ring-opening sequences involving vinyl nucleophiles. We anticipated that a chiral catalyst such as one of the ketones developed by Shi and co-workers [15–18] would control the stereochemistry of the epoxidation of **6**. Presumably, the identity of the protecting groups on this substrate (i.e., R^2^ and R^3^) would be critical to the success of the reaction. After formation of the epoxide, the bulky trityl ether was envisioned to direct the subsequent ring-opening to the distal carbon [19–22]. Alkene **6** would ultimately be formed by a Myers alkylation [23] of (+)-pseudoephedrine derived amide **7** with allylic iodide **8**.

**Scheme 2:**
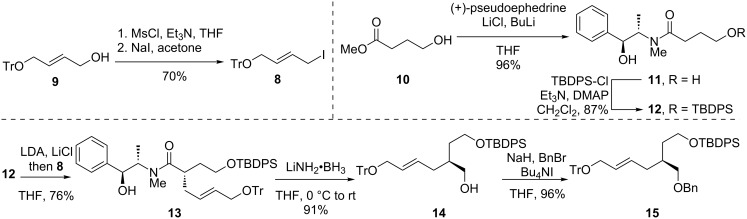
Synthesis of triether **15**.

The initial epoxidation substrate of type **6** that we targeted possessed benzyl and TBDPS ethers as the protecting groups. First, allylic iodide **8** was synthesized by iodination of the mesylate derived from known alcohol **9** [24] (Scheme 2). Then, coupling of methyl γ-hydroxybutyrate (**10**) [25] with lithiated (+)-pseudoephedrine afforded amide **11** in excellent yield. Selective silylation of the primary alcohol of **11** delivered substrate **12**. Alkylation of the enolate derived from **12** with **8** according to the Myers protocol [23] furnished adduct **13** in very high yield. Although not measured directly, the dr of this compound was assumed to be very high (i.e., ≥95:5) based on the results of an alkylation conducted on a very similar substrate (see below). The configuration of the newly formed stereocenter of **13** was assigned based on the established stereochemical course of the Myers alkylation [23]. Finally, reductive removal of the chiral auxiliary with lithium amidotrihydroborate [26] produced alcohol **14**, and benzylation yielded triether **15**.

**Scheme 3:**
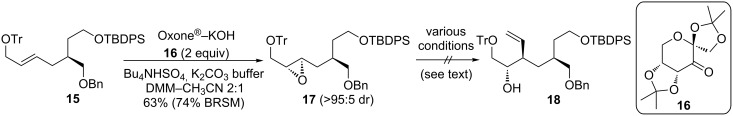
Synthesis and attempted ring-opening of epoxide **17**.

**Scheme 4:**
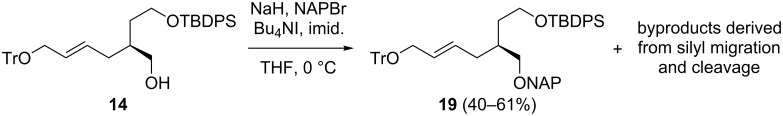
Attempted protection of **14** and silyl migration.

Asymmetric epoxidation of alkene **15** was somewhat sluggish and required superstoichiometric amounts of Shi’s fructose-derived ketone **16** [27]. The resulting epoxide **17** was produced in moderate yield but excellent (<95:5) diastereomeric ratio (Scheme 3). The epoxide stereochemistry was assigned based on the reported outcomes of epoxidations mediated by **16** [27]. Epoxide **17** was then subjected to ring-opening reactions with vinyl Grignard reagents in the presence of various copper salts. Surprisingly, only trace amounts of the desired product were detected, with recovered starting material and multiple byproducts typically comprising the majority of the mass balance. Although not investigated in detail, analysis of these reactions by ^1^H NMR and mass spectrometry indicated that partial debenzylation was occurring. Accordingly, we decided to replace the benzyl ether with a 2-naphthylmethyl (NAP) ether [28]. Unfortunately, attempted protection of alcohol **14** produced varying yields of triether **19** along with byproducts derived from migration and/or scission of the TBDPS ether (Scheme 4). Modification of the reaction conditions failed to suppress the deleterious silyl migration and cleavage.

**Scheme 5:**
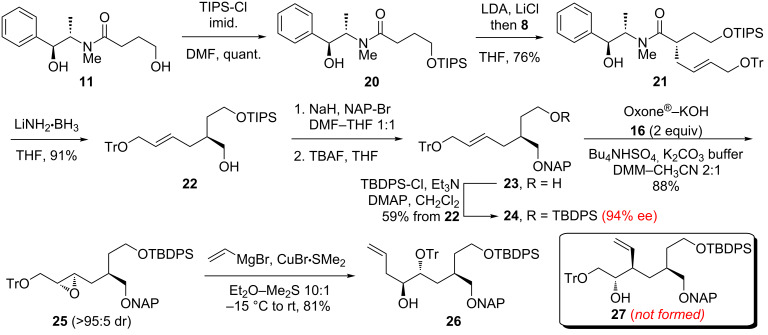
Synthesis and ring-opening rearrangement of epoxide **25**.

With the hope that a more robust silyl ether would not migrate, we installed a TIPS group on alcohol **11** (Scheme 5). Gratifyingly, TIPS-protected amide **20** was alkylated by **8** in the same yield as TBDPS-protected amide **12**. Reductive removal of the chiral auxiliary furnished alcohol **22** in 91% yield. Fortunately, naphthylmethylation of **22** was achieved without migration of the TIPS group. Surprisingly, Shi epoxidation of the NAP ether derivative of **22** was low-yielding (<10%), and ring-opening of the resulting epoxide did not proceed. These results prompted us to swap the bulky TIPS moiety for a smaller TBDPS group, and triether **24** was obtained uneventfully in 77% overall yield from **22**. Notably, the high (94%) ee of **24** as established by chiral HPLC analysis demonstrated that the Myers alkylation of **20** had proceeded with excellent diastereoselectivity. Then, we were pleased to find that Shi epoxidation of **24** provided **25** in reasonable (72%) yield and high (>95:5) dr. After considerable experimentation, we discovered that CuBr•Me_2_S [29] in conjunction with vinylmagnesium bromide was uniquely effective at facilitating the ring-opening of **25**. However, careful inspection of the ^1^H NMR spectrum revealed the presence of one less hydrogen atom than expected in the 3–4 ppm region and one more hydrogen atom than expected in the 1–2 ppm region. Clearly, neither the anticipated product **27** nor the regioisomer derived from attack at the more hindered epoxide carbon had been generated. Instead, the NMR data were consistent with the formation of a different regioisomer, tentatively identified as alcohol **26**, which had been produced in good yield.

**Scheme 6:**
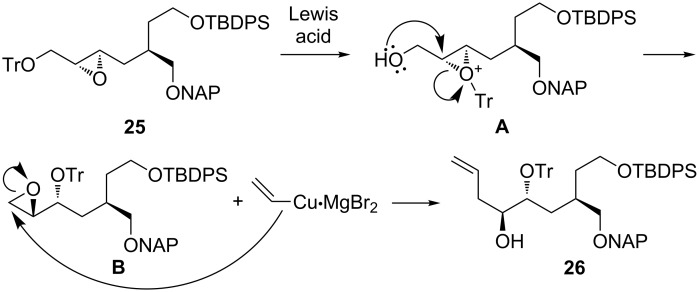
Proposed mechanism for generation of alcohol **26**.

Presumably, the extremely hindered nature of internal epoxide **25** precluded its direct ring-opening, allowing alcohol **26** to form by means of a Payne rearrangement [30]. A possible mechanistic pathway for this transformation is given in Scheme 6. Coordination of a Lewis acid (likely a copper or magnesium species) to the trityl ether moiety of **25** could promote migration of the trityl group [31–32] to the epoxide, generating intermediate **A**. Payne rearrangement of **A** would then furnish epoxide **B**. Finally, attack of the vinylcopper complex [29] at the less-hindered carbon of the epoxide would provide **26**. Acid- and Lewis acid promoted Payne rearrangements of epoxy alcohols [33–34] and epoxy methyl ethers [35] have been described, but we are unaware of any prior reports of Payne rearrangements of the bulkier epoxy trityl ethers. However, previous observations of trityl migration [31–32], although rare, do lend support to our mechanistic proposal. The NMR data for **26**, while strongly supportive of the carbon backbone as drawn, do not permit an unambiguous assignment of the trityl ether to the C4 or C5 oxygen atom. An alternative pathway to this carbon skeleton involving a Payne rearrangement without trityl migration can also be envisioned, and under this scenario, the trityl ether would be located at C4 rather than C5. This possibility cannot be ruled out, but it would require opening of an activated epoxonium species at the less-substituted carbon instead of the more-substituted carbon as is typically observed. Thus, we favor the mechanism shown in Scheme 6.

**Scheme 7:**
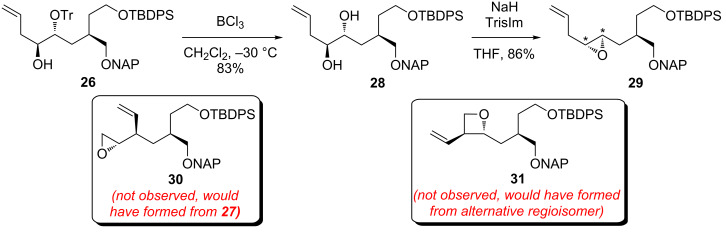
Synthesis of epoxide **29** from alcohol **26** (asterisks indicate relative but not absolute stereochemistry).

To provide additional evidence for the structure of **26**, this compound was converted into epoxide **29** as outlined in Scheme 7. Selective detritylation was accomplished by exposure to BCl_3_ at low temperature [36]. Camphorsulfonic acid was also effective for this transformation, although lengthy reaction times were required. Treatment of the resulting diol **28** with 2,4,6-triisopropylbenzenesulfonyl imidazole (TrisIm) effected regioselective sulfonylation (presumably of the less-hindered homoallylic alcohol, although this cannot be known for sure) followed by cyclization [37], delivering a single *trans*-disubstituted epoxide **29** of uncertain absolute stereochemistry in good yield. Examination of the ^1^H NMR spectrum of **29** clearly demonstrated that a disubstituted epoxide had been generated. Alcohol **27**, or the aforementioned regioisomer that would have resulted from ring-opening of epoxide **25** at the more hindered carbon, would have afforded terminal epoxide **30** or oxetane **31**, respectively, when subjected to this two-step sequence. While these observations do not shed light on the location of the trityl ether in **26**, they do provide compelling evidence that the carbon backbone of this compound is correct as drawn and is produced by a Payne rearrangement of some type.

## Conclusion

In the context of synthetic efforts targeting the polycyclic alkaloid lyconadin A, we prepared scalemic epoxide **25**. A Myers alkylation and a reagent-controlled Shi epoxidation were used to construct this compound in a highly stereoselective fashion. The bulky trityl group of **25** was intended to serve as a means of directing a ring-opening reaction to the distal carbon of the epoxide [19–22]. However, an unanticipated Lewis acid promoted Payne rearrangement intervened, producing alcohol **26** instead of the expected regioisomer **27**. We believe that the extremely hindered nature of epoxide **25** prevented the desired ring-opening process, thereby enabling the unusual rearrangement to proceed. Conceivably, future studies of the scope and limitations of Lewis acid promoted Payne rearrangement–ring-opening cascades could establish their utility in organic synthesis.

## Supporting Information

File 1 Name: Experimental procedures and characterization data for all new compounds.
