# Supplementary material for: Exploration of an epoxidation–ring-opening strategy for the synthesis of lyconadin A and discovery of an unexpected Payne rearrangement
Source: Beilstein J Org Chem. 2013 Jun 18;9:1179–84. doi: 10.3762/bjoc.9.132 (PMC3701374; doi:10.3762/bjoc.9.132)
Supplement: File 1 — Name: Experimental procedures and characterization data for all new compounds. [file Beilstein_J_Org_Chem-09-1179-s001.pdf]

Supporting Information

for

Exploration of an epoxidation–ring opening strategy for the synthesis of lyconadin A and discovery of an unexpected Payne rearrangement

Brad M. Loertscher, Yu Zhang, and Steven L. Castle*

Address: Department of Chemistry and Biochemistry, Brigham Young University, C100 BNSN, Provo, UT, 84602, USA

E-mail: Steven L. Castle - scastle@chem.byu.edu

*Corresponding author

Experimental procedures and characterization data for all new compounds.

Table of contents

General experimental details	S2
Experimental procedures and spectral data	S2–S10
References	S11

General experimental details

Dichloromethane, diethyl ether, dimethylformamide, tetrahydrofuran, and triethylamine were dried by passage through a solvent drying system containing cylinders of activated alumina [1]. Other solvents and reagents were purchased from commercial vendors and used without purification. Flash chromatography was carried out using 60–230 mesh silica gel. ^{1}H NMR spectra were acquired on a 500 MHz spectrometer with chloroform (7.27 ppm) as internal reference unless otherwise specified. Signals are reported as follows: s (singlet), d (doublet), t (triplet), dd (doublet of doublets), dt (doublet of triplets), ddd (doublet of doublet of doublets), br s (broad singlet), m (multiplet). Coupling constants are reported in hertz (Hz). ^{13}C NMR spectra were acquired on a spectrometer operating at 125 MHz with chloroform (77.23 ppm) as internal reference. Infrared spectra were obtained on an FT-IR spectrometer. Mass spectral data were obtained using ESI techniques.

Experimental procedures and spectral data

(*E*)-1-(Trityloxy)-4-iodobut-2-en (8). Triethylamine (5.40 mL, 3.94 g, 39.0 mmol) and methanesulfonyl chloride (1.50 mL, 2.22 g, 19.4 mmol) were added successively to a solution of alcohol **9** [2] (4.2904 g, 12.984 mmol) in anhydrous THF (100 mL) at 0 °C under Ar. The resultant mixture was warmed to rt and stirred for 4 h. The reaction was quenched with H_2O (50 mL) and extracted with EtOAc (3 × 50 mL). The combined organic layers were washed with brine (100 mL), dried (Na_2SO_4), and concentrated in vacuo. **NOTE:** *The procedure below was performed in the dark as the product is light-sensitive.* The crude mesylate was dissolved in acetone (100 mL) and treated with NaI (5.7464 g, 38.337 mmol). The resultant mixture heated under reflux in Ar for 18 h **in the dark**. The reaction was quenched by the addition of H_2O (50 mL), and the volatiles were removed in vacuo. The remaining aqueous layer was extracted with EtOAc (3 × 50 mL), and

the combined organic layers were washed with brine (100 mL), dried (Na_2SO_4), and concentrated in vacuo. Flash chromatography (500 mL of SiO_2, 0–2% EtOAc in hexanes gradient elution) afforded iodide **8** (4.0037 g, 9.0927 mmol, 70%) as a white solid: ^{1}H NMR ($CDCl_3$, 500 MHz) δ 7.46–7.42 (m, 6H), 7.31 (t, *J* = 7.6 Hz, 6H), 7.26–7.23 (m, 3H), 6.12–6.05 (m, 1H), 5.80 (dt, *J* = 15.2, 4.9 Hz, 1H), 3.91 (d, *J* = 7.8 Hz, 2H), 3.62 (d, *J* = 4.4 Hz, 2H); HRMS (ESI) *m/z* 441.0732 (MH^+, $C_{23}H_{21}OIH^+$ requires 441.0710).

4-Hydroxy-*N*-((1*S*,2*S*)-1-hydroxy-1-phenylpropan-2-yl)-*N*-methylbutanamide (11). A solution of *n*-BuLi (1.6 M in hexane, 2.10 mL, 3.36 mmol) was added to an ice-cooled suspension of LiCl (810.7 mg, 19.12 mmol) and (1*S*,2*S*)-(+)-pseudoephedrine (1.0534 g, 6.3754 mmol) in anhydrous THF (40 mL), and the suspension was stirred at 0 °C under Ar for 30 min. Then, a solution of methyl 4-hydroxybutanoate [3] (**10**, 1.5062 g, 12.750 mmol) in anhydrous THF (8 mL) was added to the mixture, and it was warmed to rt and stirred under Ar for 3 h. The reaction was quenched by the addition of 0.5 N NaOH (20 mL), and the volatiles were removed in vacuo. The residue was extracted with $CHCl_3$–iPrOH 3:1 (4 × 25 mL), and the combined organic layers were washed with brine (100 mL), dried (Na_2SO_4), and concentrated in vacuo. Flash chromatography (200 mL of SiO_2, 3–10% MeOH in CH_2Cl_2 gradient elution) afforded **11** (1.5416 g, 6.1338 mmol, 96%) as a brown oil: ^{1}H NMR ($CDCl_3$, 500 MHz, mixture of rotamers, data for major rotamer) δ 7.41–7.28 (m, 5H), 4.59 (dd, *J* = 13.8, 7.8 Hz, 1H), 4.54 (br s, 1H), 3.74–3.66 (m, 3H), 2.88 (s, 3H), 2.60 (br s, 1H), 2.55–2.41 (m, 2H), 1.93–1.88 (m, 2H), 1.09 (d, *J* =6.7 Hz, 3H); ^{13}C NMR ($CDCl_3$, 125 MHz, mixture of rotamers, data for major rotamer) δ 173.8, 142.2, 127.5 (2C), 126.7, 126.1 (2C), 75.0, 61.9, 57.7, 29.7, 27.8, 26.4, 13.6; IR (film) ν_{max} 3376, 2937, 1614, 1482, 1453, 1407, 1049 cm^{-1}; HRMS (ESI) *m/z* 274.14082 (MNa^+, $C_{14}H_{21}NO_3Na^+$ requires 274.14136).

***N*-((1*S*,2*S*)-1-hydroxy-1-phenylpropan-2-yl)-*N*-methyl-4-((triisopropylsilyl)oxy) butanamide (20).** A solution of **11** (294.5 mg, 1.172 mmol) in anhydrous DMF (1.6 mL) at rt under Ar was treated with imidazole (199.4 mg, 2.929 mmol) and TIPS-Cl (390 μL, 351 mg, 1.82 mmol). The resultant mixture was stirred at rt for 24 h, and the reaction was quenched by the addition of brine (10 mL). The mixture was extracted with CH_2Cl_2 (3 × 10 mL), and the combined organic layers were washed with brine (30 mL), dried (Na_2SO_4), and concentrated in vacuo. Flash chromatography (50 mL of SiO_2, 5% MeOH in CH_2Cl_2 elution) afforded **20** (477.7 mg, 1.172 mmol, quant.) as a yellow oil: ^{1}H NMR ($CDCl_3$, 500 MHz, mixture of rotamers, data for major rotamer) δ 7.37–7.29 (m, 5H), 4.59–4.54 (m, 1H), 4.50 (br s, 1H), 3.75–3.69 (m, 3H), 2.93 (s, 3H), 2.36 (m, 2H), 1.86–1.73 (m, 2H), 1.06–0.96 (m, 24H); ^{13}C NMR ($CDCl_3$, 125 MHz, mixture of rotamers, data for major rotamer) δ 175.3, 142.6, 128.3 (2C), 127.6, 126.5 (2C), 76.4, 62.4, 58.3, 30.6, 28.4, 26.8, 18.0 (6C), 14.4, 12.0 (3C); IR (film) ν_{max} 3388, 2942, 2865, 1623, 1463, 1405, 1105, 1067 cm^{-1}; HRMS (ESI) *m/z* 430.27546 (MNa^+, $C_{23}H_{41}NO_3SiNa^+$ requires430.27479).

(*S*,*E*)-*N*-((1*S*,2*S*)-1-Hydroxy-1-phenylpropan-2-yl)-*N*-methyl-2-(2-(triisopropyl-silyloxy)ethyl)-6-(trityloxy)hex-4-enamide (21). A solution of *n*-BuLi (1.6 M in hexane, 1.50 mL, 2.40 mmol) was added to a suspension of LiCl (flame-dried, 294.9 mg, 6.956 mmol) and *i*-Pr_2NH (370 μL, 267 mg, 2.64 mmol) in anhydrous THF (2.8 mL) at –78 °C under Ar. The resulting suspension was stirred at –78 °C under Ar for 10 min, then warmed to 0 °C, stirred for 5 min, cooled to –78 °C, and treated with an ice-cooled solution of **20** (472.6 mg, 1.159 mmol) in anhydrous THF (1.4 mL). The mixture was stirred at –78 °C under Ar for 1 h, at 0 °C for 15 min, and at rt for 5 min. It was then cooled to 0 °C and treated with allylic iodide **8** (578.9 mg, 1.3147 mmol). The resultant mixture was stirred at 0 °C under Ar for 2.5 h. The reaction

was quenched by the addition of sat aq NH_4Cl (18 mL) and H_2O (18 mL), and the mixture was extracted with EtOAc (3 × 40 mL). Flash chromatography (SiO_2, 1% MeOH in CH_2Cl_2 elution) afforded **21** (609.1 mg, 0.8459 mmol, 73%) as a white solid: ^{1}H NMR ($CDCl_3$, 500 MHz, mixture of rotamers) δ 7.45–7.41 (m, 3H), 7.38–7.20 (m, 17H), 5.74–5.60 (m, 2H), 4.65–4.62 (m, 1H), 4.48 (br s, 1H), 3.81–3.77 (m, 2H), 3.69–3.65 (m, 1H), 3.60–3.52 (m, 2H), 3.13–3.09 (m, 1H), 2.97 and 2.95 (2s, 3H), 2.42–2.36 (m, 1H), 2.25–2.20 (m, 1H), 1.93–1.86 (m, 1H), 1.78–1.69 (m, 1H), 1.17–1.05 (m, 24H); ^{13}C NMR ($CDCl_3$, 125 MHz) δ 178.0, 144.5 (3C), 142.9, 131.1, 129.1 (2C), 128.9 (2C), 128.6, 128.5 (6C), 128.0 (6C), 127.7, 127.2 (3C), 87.0, 76.4, 64.9, 61.1, 60.8, 58.1, 38.6, 36.1, 35.7, 18.4 (6C), 14.9, 12.3 (3C); IR (film) ν_{max} 3388, 2942, 2865, 1623, 1463, 1405, 1366, 1105 cm^{-1}; HRMS (ESI) *m/z* 742.42604 (MNa^+, $C_{46}H_{61}NO_4SiNa^+$ requires 742.42621).

(*S*,*E*)-2-(2-(Triisopropylsilyloxy)ethyl)-6-(trityloxy)hex-4-en-1-ol (22). A flame-dried round bottom flask containing anhydrous THF (200 mL) at –78 °C under Ar was treated with iPr_2NH (10.3 mL, 7.44 g, 72.97 mmol) followed by *n*-BuLi (1.6 M in hexanes, 42.0 mL, 67.2 mmol). The resultant mixture was stirred at –78 °C for 10 min, at 0 °C for 5 min, and then cooled to –78 °C. Borane–ammonia complex (90%, 2.38 g, 69.4 mmol) was added to the mixture in one portion, and it was stirred at 0 °C for 20 min, then at rt for 20 min. The mixture was then cooled to 0 °C and treated dropwise with a solution of **21** (12.5 g, 17.4 mmol) in anhydrous THF (127 mL). The resulting mixture was stirred at rt under Ar for 50 min, then cooled to 0 °C and treated with sat aq NH_4Cl (50 mL). The aqueous layer was extracted with EtOAc (3 × 50 mL), and the combined organic layers were washed with brine (25 mL), dried (Na_2SO_4), and concentrated in vacuo. Flash chromatography (SiO_2, 20% EtOAc in hexanes elution) afforded **22** (8.83 g, 15.8 mmol, 91%) as a colorless oil: ^{1}H NMR ($CDCl_3$, 500 MHz) δ 7.55 (d, *J* = 7.0 Hz, 6H), 7.36 (t,

J = 7.5 Hz, 6H), 7.28 (t, J = 7.0 Hz, 3H), 5.84–5.78 (m, 1H), 5.74–5.69 (m, 1H), 3.97–3.93 (m,1H), 3.86–3.81 (m, 1H), 3.75–3.69 (m, 1H), 3.66 (d, J = 5.5 Hz, 2H), 3.60–3.56 (m, 1H), 3.49 (br s, 1H), 2.63–2.21 (m, 1H), 2.17–2.09 (m, 1H), 1.88–1.84 (m, 1H), 1.82–1.76 (m, 1H), 1.69–1.63 (m, 1H), 1.18–1.16 (m, 21H); ^{13}C NMR ($CDCl_3$, 125 MHz) δ 144.6 (3C), 130.8, 129.0 (7C), 128.1 (6C), 127.2 (3C), 87.1, 66.2, 65.2, 62.5, 39.9, 35.6, 35.3, 18.3 (6C), 12.2 (3C); IR (film) ν_{max} 3415, 2941, 2865, 1490, 1448, 1381, 1098, 1055 cm^{-1}; HRMS (ESI) *m/z* 576.38523 $(M(NH_4)^+$, $C_{36}H_{50}O_3SiNH_4^+$ requires 576.38675).

(*S*,*E*)-*tert*-Butyl((3-((naphthalen-2-ylmethoxy)methyl)-7-(trityloxy)hept-5-en-1-yl)oxy)diphenylsilane (24). A solution of **22** (500 mg, 0.895 mmol) in anhydrous DMF–THF 1:1 (20 mL) at 0 °C under Ar was treated with NaH (60% dispersion in mineral oil, 72.0 mg, 1.80 mmol) followed by 2-(bromomethyl)naphthalene (297 mg, 1.34 mmol). The resultant mixture was stirred at 0 °C under Ar for 12 h, then treated with sat aq NH_4Cl (6 mL) and extracted with EtOAc (3 × 10 mL). The combined organic layers were washed with brine (5 mL), dried (Na_2SO_4), and concentrated in vacuo. Flash chromatography (SiO_2, 2% EtOAc in hexanes elution) afforded the triether (557 mg, 0.797 mmol, 89%) as a colorless oil.

A solution of the obtained triether (557 mg, 0.797 mmol) in anhydrous THF (15 mL) at 0 °C under Ar was treated with TBAF (1.0 M in THF, 1.80 mL, 1.80 mmol). The resultant mixture was stirred at rt under Ar for 8 h. then treated with sat aq NH_4Cl (4 mL) and extracted with EtOAc (3 × 8 mL). The combined organic layers were washed with brine (5 mL), dried (Na_2SO_4), and concentrated in vacuo. Flash chromatography (SiO_2, 35% EtOAc in hexanes elution) afforded alcohol **23** (411 mg, 0.757 mmol, 95%) as a colorless oil.

A solution of **23** (411 mg, 0.757 mmol) in anhydrous CH_2Cl_2 (6 mL) at 0 °C under Ar was treated with Et_3N (150 μL, 109 mg, 1.08 mmol), DMAP (22.0 mg, 0.180 mmol), and TBDPS-Cl

(260 μL, 275 mg, 1.00 mmol). The resultant mixture was stirred at rt under Ar for 24 h. The reaction was quenched by the addition of sat aq NH_4Cl (3 mL), and the layers were separated. The aqueous layer was extracted with EtOAc (3 × 5 mL), and the combined organic layers were washed with brine (4 mL), dried (Na_2SO_4), and concentrated in vacuo. Flash chromatography (SiO_2, 2% EtOAc in hexanes elution) afforded **24** (411.8 mg, 0.527 mmol, 70% as a colorless oil: ^{1}H NMR ($CDCl_3$, 500 MHz) δ 7.85–7.76 (m, 4H), 7.67–7.65 (m, 4H), 7.48–7.21 (m, 24H), 5.71–5.66 (m,1H), 5.64–5.59 (m, 1H), 4.64 (s, 2H), 3.76 (dt, *J* = 6.6, 1.5 Hz, 2H), 3.54 (d, *J* = 5.0 Hz, 2H), 3.45–3.38 (m, 2H), 2.27–2.23 (m, 1H), 2.18–2.14 (m, 1H), 2.02–1.98 (m, 1H), 1.71–1.63 (m, 2H), 1.07 (s, 9H); ^{13}C NMR ($CDCl_3$, 125 MHz) δ 144.5 (3C), 136.3, 135.7 (2C), 134.1, 133.4, 133.0, 130.6, 129.7 (2C), 128.8 (4C), 128.7, 128.19, 128.16, 128.0, 127.9 (4C), 127.8, 127.7 (6C), 127.0 (6C), 126.3, 126.1, 125.8 (3C), 73.2, 73.0, 65.1, 62.2, 60.5, 35.5, 34.6, 34.1, 27.0 (3C), 19.3; IR (film) ν_{max} 3055, 2929, 2856, 1489, 1448, 1428, 1372, 1265, 1110 cm^{-1}; HRMS (ESI) *m/z* 798.43376 ($M(NH_4)^+$, $C_{54}H_{56}O_3SiNH_4^+$ requires 798.43370). **24** was obtained in 94% *ee* as analyzed by HPLC (Chiralcel OD-H, 99.2:0.8 hexane:*i*-PrOH, 1 mL/min; t_R = 6.7 min (major), 7.9 min (minor)).

***tert*-Butyl((*R*)-4-((naphthalen-2-ylmethoxy)methyl)-3-(((2*R*,3*R*)-3-(trityloxymethyl)-oxiran-2-yl)methyl)butoxy)diphenylsilane (25).** A solution of alkene **24** (128.6 mg, 0.1646 mmol) in dimethoxymethane/CH_3CN (2:1, 1.86 mL) was treated sequentially with a K_2CO_3–CH_3COOH buffer solution [4] (1.12 mL), Bu_4NHSO_4 (1.5 mg, 0.0044 mmol), and ketone **16** (52.0 mg, 0.201 mmol). A solution of Oxone® (161.2 mg, 0.2622 mmol) in 4 × 10^{-4} M aq Na_2EDTA (0.62 mL) and a 1.47 M aq KOH solution (0.62 mL) were added simultaneously and dropwise to this mixture at the same rate. The resulting suspension was stirred at rt for 5 h, and an additional quantity of ketone **16** (52.0 mg, 0.201 mmol) was added. Then, a solution of

Oxone® (161.2 g, 0.2622 mmol) in 4 × 10^{-4} M aq Na_2EDTA (0.62 mL) and a 1.47 M aq KOH solution (0.62 mL) were once again added simultaneously and dropwise to this mixture at the same rate. The resulting suspension was stirred at rt for 4 h and then extracted with EtOAc (3 × 5 mL). The combined organic layers were washed with brine (3 mL), dried (Na_2SO_4), and concentrated in vacuo. Flash chromatography (SiO_2, 1% EtOAc in hexanes elution) afforded **25** (115.0 mg, 0.144 mmol, 88%) as a colorless oil: ^1H NMR ($CDCl_3$, 500 MHz) δ 7.83–7.71 (m, 4H), 7.65–7.62 (m, 4H), 7.48–7.44 (m, 8H), 7.41–7.21 (m, 16H), 4.60 (s, 2H), 3.72 (t, *J* = 6.5 Hz, 2H), 3.45 (d, *J* = 5.5 Hz, 2H), 3.23 (dd, *J* = 10.0, 3.5 Hz, 1H), 3.09 (dd, *J* = 11.0, 6.0 Hz, 1H), 2.90–2.88 (m, 1H), 2.82–2.81 (m, 1H), 2.13–2.08 (m, 1H), 1.71–1.56 (m, 4H), 1.02 (s, 9H); ^{13}C NMR ($CDCl_3$, 125 MHz) δ 143.9 (3C), 136.0, 135.5 (2C), 133.8, 133.2, 132.9, 129.6 (2C), 128.7 (4C), 128.1 (4C), 127.9, 127.8 (6C), 127.7, 127.6 (6C), 127.0 (3C), 126.2, 126.0, 125.75, 125.67, 86.7, 73.10, 73.08, 64.7, 61.8, 57.2, 55.0, 34.2, 34.1, 33.8, 26.9 (3C), 19.2; IR (film) ν_{max} 2928, 1448, 1427, 1110 cm^{-1}; HRMS (ESI) *m/z* 814.43165 ($M(NH_4)^+$, $C_{54}H_{56}O_4SiNH_4^+$ requires 814.42861).

(4*S*,5*R*,7*R*)-9-(*tert*-Butyldiphenylsilyloxy)-7-((naphthalen-2-ylmethoxy)methyl)-5-(trityloxy)non-1-en-4-ol (26). A mixture of CuBr•Me_2S (23.2 mg, 0.113 mmol) and Me_2S (51 µL) in anhydrous Et_2O (0.51 mL) at –15 °C under Ar was treated with vinylmagnesium bromide (1.0 M in THF, 0.34 mL, 0.34 mmol). The resultant mixture was stirred at –15 °C for 30 min and at 0 °C for 30 min. A solution of epoxide **25** (45.0 mg, 0.0565 mmol) in anhydrous Et_2O (200 µL) was added dropwise to the mixture. The resultant mixture was warmed to rt and stirred under Ar for 18 h. The reaction was quenched by the addition of brine (0.5 mL). The mixture was extracted with EtOAc (3 × 3 mL), and the combined organic layers were washed with brine (1 mL), dried (Na_2SO_4), and concentrated in vacuo. Flash chromatography (SiO_2, 5% EtOAc in hexanes elution)

afforded **26** (37.8 mg, 0.0458 mmol, 81%) as a colorless oil: ^{1}H NMR ($CDCl_3$, 500 MHz) δ 7.84–7.65 (m, 8H, Ar-H), 7.50–7.43 (m, 9H, Ar-H), 7.41–7.23 (m, 15H, Ar-H), 5.77 (ddd, *J* = 17.4 Hz, 10.7 Hz, 8.8 Hz, 1H, C2-H), 5.31 (ddd, *J* = 17.4 Hz, 5.4 Hz, 1.5 Hz, 1H, C1-H), 5.18 (ddd, *J* = 10.7 Hz, 6.3 Hz, 1.5 Hz, 1H, C1-H), 4.58 (s, 2H, -O$\underline{CH_2}$Ar), 3.73–3.67 (m, 2H, C9-Ha,b), 3.38–3.30 (m, 2H, -$\underline{CH_2}$ONAP), 3.10 (dd, *J* = 8.5 Hz, 1.5 Hz, 1H, C4-H or C5-H), 3.04 (t, *J* = 8.1 Hz, 1H, C4-H or C5-H), 2.36 (d, *J* = 5.9 Hz, 1H, -OH), 1.75–1.73 (m, 1H, C3-Ha), 1.62–1.52 (m, 3H, C3-Hb and C6- or C8-Ha,b), 1.44–1.38 (m, 1H, C7-H), 1.37–1.27 (m, 2H, C6- or C8-Ha,b), 1.02 (s, 9H, -SiC$(CH_3)_3$); ^{13}C NMR ($CDCl_3$, 125 MHz) δ 144.0 (3C), 142.0, 136.4, 135.8 (2C), 134.3, 133.5, 133.2, 129.7 (2C), 129.0 (4C), 128.2 (2C), 128.0 (4C), 127.9 (6C), 127.8 (6C), 127.3 (2C), 126.4, 126.2, 125.9 (3C), 114.2, 75.5, 73.6, 73.5, 73.3, 69.7, 62.3, 35.6, 35.0, 34.8, 34.7, 27.1 (3C), 19.4; IR (film) ν_{max} 3056, 2929, 2857, 1599, 1489, 1448, 1427, 1088, 1032 cm^{-1}; HRMS (ESI) *m/z* 842.45747 (MNH_4^+, $C_{56}H_{60}O_4SiNH_4^+$ requires 842.45991).

(4*S*,5*R*,7*R*)-9-(*tert*-Butyldiphenylsilyloxy)-7-((naphthalen-2-ylmethoxy)methyl)non-1-ene-4,5-diol (28). A solution of BCl_3 (1.0 M in CH_2Cl_2, 65 μL, 0.065 mmol) was added dropwise to a solution of alcohol **26** (54.9 mg, 0.0665 mmol) in anhydrous CH_2Cl_2 (2.0 mL) at –35 ºC under Ar. The resultant mixture was stirred at –35 ºC for 30 min, and the reaction was quenched by the addition of MeOH (2.0 mL). The suspension was poured into sat aq $NaHCO_3$ (5 mL) and extracted with CH_2Cl_2 (3 × 5 mL). The combined organic layers were washed with brine (10 mL), dried (Na_2SO_4), and concentrated in vacuo. Flash chromatography (10 mL of SiO_2, 20% EtOAc in hexanes elution) afforded diol **28** (32.1 mg, 0.0551 mmol, 83%) as a colorless oil: ^{1}H NMR ($CDCl_3$, 500 MHz) δ 7.86–7.75 (m, 4H), 7.68–7.66 (m, 4H), 7.50–7.48 (m, 2H), 7.45–7.41 (m, 3H), 7.39–7.37 (m, 4H), 5.73 (dd, *J* = 17.1 Hz, 10.7 Hz, 1H), 5.33 (dd, *J* = 17.1 Hz, 5.1 Hz, 1H), 5.24 (dd, *J* = 10.7 Hz, 1.1 Hz, 1H), 4.61 (s, 2H), 3.74–3.70 (m, 2H), 3.45–3.40 (m, 3H), 3.35 (dd, *J*

= 9.3 Hz, 6.3 Hz, 1H), 2.30 (d, *J* = 19.5 Hz, 1H), 1.87–1.85 (m, 2H), 1.64–1.54 (m, 3H), 1.47–1.42 (m, 3H), 1.06 (s, 9H); ^{13}C NMR ($CDCl_3$, 125 MHz) δ 140.6, 136.0, 135.6 (4C), 134.0 (2C), 133.3, 133.0, 129.6 (2C), 128.1, 127.9, 127.7, 127.6 (4C), 126.3, 126.1, 125.83, 125.78, 115.3, 76.0, 73.1, 68.9, 68.8, 62.0, 35.4, 34.5, 34.0, 26.9 (3C), 25.0, 19.2; IR (film) ν_{max} 3418, 2930, 2857, 1471, 1427, 1389, 1110 cm^{-1}; HRMS (ESI) *m/z* 605.30492 (MNa^+, $C_{37}H_{46}O_4SiNa^+$ requires 605.30576).

((*R*)-4-((2*R*,3*R*)-3-Allyloxiran-2-yl)-3-((naphthalen-2-ylmethoxy)methyl)butoxy)(*tert*-butyl)diphenylsilane (29). A solution of **28** (13.5 mg, 0.0232 mmol) in anhydrous THF (1.0 mL) at 0 °C under Ar was treated with NaH (60% dispersion in mineral oil, 1.9 mg, 0.0475 mmol). The resultant mixture was stirred at rt for 30 min, then cooled to 0 °C and treated with *N*-(2,4,6-triisopropylbenzenesulfonyl)imidazole (8.5 mg, 0.0254 mmol). The mixture was stirred at rt for 2 h, treated with sat aq NH_4Cl (5 mL), and extracted with EtOAc (3 × 5 mL). The combined organic layers were washed with brine (5 mL), dried (Na_2SO_4), and concentrated in vacuo. Flash chromatography (SiO_2, 6% EtOAc in hexanes elution) afforded **29** (11.3 mg, 0.0200 mmol, 86%) as a colorless oil: ^{1}H NMR ($CDCl_3$, 500 MHz) δ 7.86–7.75 (m, 4H), 7.68–7.66 (m, 4H), 7.49 (t, *J* = 4.5 Hz, 2H), 7.45–7.43 (m, 3H), 7.38 (t, *J* = 7.0 Hz, 4H), 5.77–5.71 (ddd, *J* = 17.5 Hz, 10.8 Hz, 3.3 Hz, 1H), 5.33 (d, *J* = 17.5 Hz, 1H), 5.20 (d, *J* = 10.5 Hz, 1H), 4.62 (s, 2H), 3.75–3.71 (m, 2H), 3.44–3.35 (m, 2H), 2.77 (dd, *J* = 7.3 Hz, 5.3 Hz, 1H), 2.66–2.65 (m, 1H), 1.89–1.87 (m, 1H), 1.75–1.52 (m, 4H), 1.51–1.44 (m, 1H), 1.30–1.26 (m, 1H), 1.06 (s, 9H); ^{13}C NMR ($CDCl_3$, 125 MHz) δ 137.54, 137.47, 135.6 (2C), 134.0, 133.3, 132.9, 129.6 (4C), 128.1, 127.9, 127.7, 127.6 (4C), 126.2, 126.1, 125.8, 125.7 (2C), 116.5, 73.1, 73.03, 72.96, 62.0, 55.0, 54.9, 35.2, 35.1, 34.4, 26.9 (3C), 19.2; IR (film) ν_{max} 2929, 2856, 2359, 1472, 1428, 1362, 1111 cm^{-1}; HRMS (ESI) *m/z* 582.3411 ($M(NH_4)^+$, $C_{37}H_{44}O_3SiNH_4$ requires 582.3398).

References

1. Pangborn, A. B.; Giardello, M. A.; Grubbs, R. H.; Rosen, R. K.; Timmers, F. J. *Organometallics* **1996**, *15*, 1518–1520.

2. Hernandez, A.-I.; Balzarini, J.; Karlsson, A.; Camarasa, M.-J.; Perez-Perez, M.-J. *J. Med. Chem.* **2002**, *45*, 4254–4263.

3. Gannett, P. M.; Nagel, D. L.; Reilly, P. J.; Lawson, T.; Sharpe, J.; Toth, B. *J. Org. Chem.* **1988**, *53*, 1064–1071.

4. Wang, Z.-X.; Shu, L.; Frohn, M.; Tu, Y.; Shi, Y. *Org. Synth.* **2009**, *Coll. Vol. 11*, 183–188.
